# Activating *BRAF* mutation in sclerosing mucoepidermoid carcinoma with eosinophilia of the thyroid gland: two case reports and review of the literature

**DOI:** 10.1186/s13256-019-2288-0

**Published:** 2019-12-28

**Authors:** Jasmine S. Sukumar, Senthil Sukumar, Darshana Purohit, Brian J. Welch, Jyoti Balani, Shirley Yan, Sumitha S. Hathiramani

**Affiliations:** 1 Department of Internal Medicine, UT Southwestern Medical Center, 5323 Harry Hines Boulevard, Dallas, TX 75390 USA; 2 Department of Endocrinology, Capital Diabetes and Endocrine Associates, 5801 Allentown Road, Suite 500, Camp Springs, MD 20746 USA; 3 Department of Endocrinology, Baylor University Medical Center, 3500 Gaston Avenue, Dallas, TX 75246 USA; 4 Department of Pathology, UT Southwestern Medical Center, 5323 Harry Hines Boulevard, Dallas, TX 75390 USA; 5 Division of Endocrinology, VA North Texas Healthcare System, 4500 South Lancaster Road, Dallas, TX 75216 USA

**Keywords:** Thyroid cancer, Sclerosing mucoepidermoid carcinoma with eosinophilia, BRAF, V600E, Renal metastases

## Abstract

**Background:**

Sclerosing mucoepidermoid carcinoma with eosinophilia is a rare form of thyroid carcinoma. The underlying molecular mechanisms of sclerosing mucoepidermoid carcinoma with eosinophilia tumorigenesis remain unknown.

**Case presentation:**

We present two cases of sclerosing mucoepidermoid carcinoma with eosinophilia, both with a concurrent papillary thyroid carcinoma. Patient 1, a 70-year-old Caucasian woman, presented with sclerosing mucoepidermoid carcinoma with eosinophilia with distant renal metastasis and coexisting papillary thyroid carcinoma. Patient 2, a 74-year-old Caucasian woman with a remote history of thyroid cancer treated with thyroidectomy, presented with locoregionally invasive sclerosing mucoepidermoid carcinoma with eosinophilia and recurrent papillary thyroid carcinoma in the thyroid bed. *BRAF* mutation studies were performed on the sclerosing mucoepidermoid carcinoma with eosinophilia tumors. In both cases, sclerosing mucoepidermoid carcinoma with eosinophilia was positive for the *BRAF* V600E mutation by polymerase chain reaction. Patient 1 is the first reported case of sclerosing mucoepidermoid carcinoma with eosinophilia with renal metastasis, to the best of our knowledge.

**Conclusions:**

Our findings suggest, for the first time, to our knowledge, involvement of the RAS-RAF-MEK-ERK signaling pathway in the pathogenesis of sclerosing mucoepidermoid carcinoma with eosinophilia. Thus, BRAF inhibitors may prove to be a useful targeted medical therapy in the treatment of a subset of patients with aggressive sclerosing mucoepidermoid carcinoma with eosinophilia tumors who exhibit *BRAF* activating mutation.

## Background

Sclerosing mucoepidermoid carcinoma with eosinophilia (SMECE) is a rare subtype of thyroid carcinoma of adults first reported in 1991 [1]. It is more common in women, occurs between ages 58 and 71 years old, and almost always occurs in a background of lymphocytic thyroiditis [2]. SMECE is characterized morphologically by extensive sclerosis and squamous and glandular differentiation with inflammatory infiltrate rich in eosinophils. Although SMECE shares several morphologic features with mucoepidermoid carcinoma (MEC), including squamous and glandular differentiation, MEC has noninflamed stroma devoid of eosinophilic infiltration [3]. Furthermore, on immunohistochemistry, MEC stains positive for thyroglobulin, whereas SMECE is usually positive for cytokeratin (CK) and mucin but negative for thyroglobulin and calcitonin. Positive staining for carcinoembryonic antigen (CEA) and p63 has also been reported in SMECE [1].

Clinically, SMECE often behaves in an indolent manner, but aggressive cases have been reported [1, 4]. It can be locoregionally invasive in the neck, though distant metastases have also been described. Surgical resection, based on the extent of invasion of the tumor, is currently the therapy of choice. Other treatment modalities that have been used with limited benefit include external beam radiation, traditional chemotherapy (such as carboplatin, doxorubicin, paclitaxel, and methotrexate), and radioactive iodine [1, 5–7].

Little is known about the underlying molecular mechanisms of SMECE tumorigenesis [2]. A recent study demonstrated that SMECE did not harbor mutations and translocations commonly involved in thyroid carcinogenesis, indicating that SMECE is likely molecularly and morphologically distinct from other thyroid tumors.

We report two interesting cases of SMECE with concurrent papillary thyroid carcinoma (PTC), both harboring the B-Raf proto-oncogene, serine/threonine kinase (*BRAF*) V600E activating mutation in the SMECE tumor. This novel finding suggests, for the first time, to our knowledge, involvement of the RAS-RAF-MEK-ERK signaling pathway in the pathogenesis of SMECE.

Institutional review board exemption was obtained per institutional protocol prior to the reporting of these two cases.

## Case presentation

### Patient 1

A 70-year-old Caucasian woman presented with a 2-month history of dysphagia, unintentional weight loss, and hoarseness. Physical examination revealed a right-sided thyroid mass. Computed tomography (CT) showed a large right thyroid mass arising from the posterior margin, invading the cricoid cartilage, and abutting the esophagus and trachea, measuring 3 cm × 2.7 cm. Laryngoscopy revealed a paralyzed right vocal cord and a right subglottic mass. Fine-needle aspiration of the thyroid mass revealed histology consistent with PTC. Preoperative positron emission tomography (PET) did not show distant metastasis, although the finding was significant for right kidney hydronephrosis. She was taken to the operating room with intent to perform total thyroidectomy with locoregional debulking. However, intraoperative frozen pathology of the involved recurrent laryngeal nerve and a level VI lymph node were concerning for squamous cell carcinoma. Given this unexpected intraoperative diagnosis, she subsequently underwent total thyroidectomy with bilateral neck dissection and laryngopharyngectomy with sacrifice of the right and left recurrent laryngeal nerves. The patient also underwent percutaneous endoscopic gastrostomy and tracheostomy tube placement.

Final surgical pathology showed an amended report consistent with a background of lymphocytic thyroiditis, PTC in the right thyroid lobe with largest dimension 4.2 cm, and SMECE in the inferior right thyroid lobe with largest dimension 3.5 cm. The anterior margin was positive for SMECE, and the posterior margin was positive for both PTC and SMECE. A second PTC focus of 0.5 cm was noted in the left thyroid lobe (negative margins). There were 10/53 lymph nodes in the neck involved with PTC (2/7 right central neck, 5/30 right levels II–V, 1/1 tracheal node, and 2/15 left neck level II/IV). SMECE was found infiltrating the right and left recurrent laryngeal nerves, paratracheal fibrous tissue, and posterior tracheal wall with extension to the deep submucosa. By immunohistochemistry, SMECE stained negative for thyroid transcription factor-1 (TTF-1) and thyroglobulin and positive for CK7, CK AE1/AE3, CK19, and CEA. We also tested the thyroid specimen for *BRAF* V600E mutation by polymerase chain reaction (PCR), and it was found to be positive in both the PTC and SMECE tumors of the thyroid.

Three months after initial presentation, the patient received ablation with 154.2 mCi of radioactive iodine (^131^I) for treatment of the PTC. A post-therapy whole-body scan done 1 week later showed focal uptake at the midline of the lower neck consistent with residual thyroid tissue or functioning metastasis, without evidence of distant metastatic disease.

**Fig. 1:**
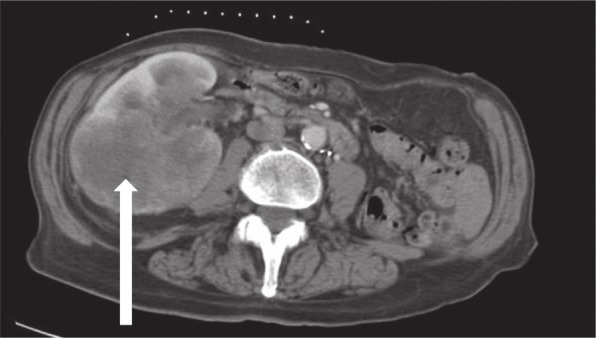
Axial computed tomography of the abdomen of patient 1 at the level of kidneys showing right renal metastases of primary thyroid sclerosing mucoepidermoid carcinoma with eosinophilia (arrow)

**Fig. 2:**
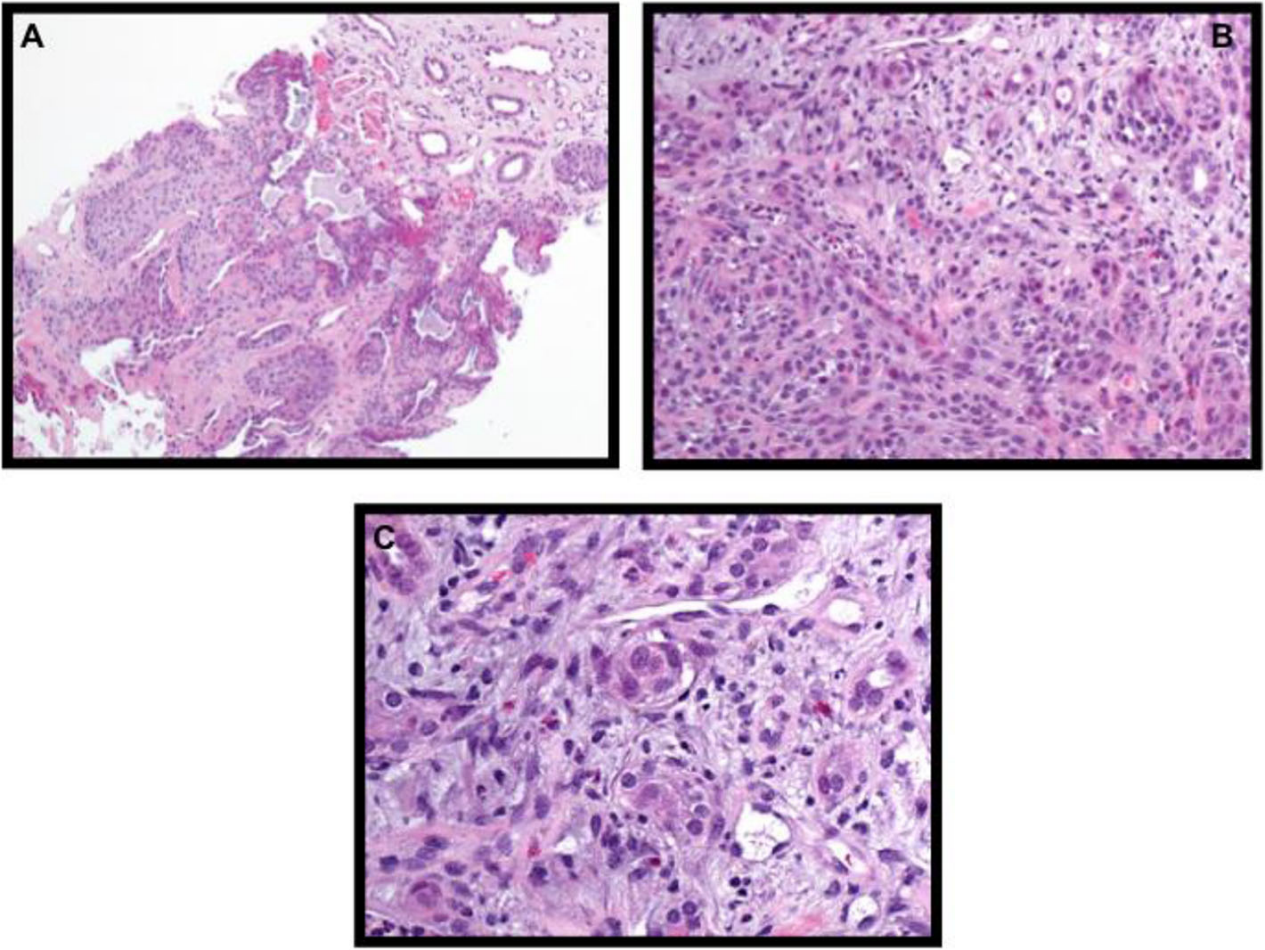
Pathology from right kidney biopsy of patient 1 shows sclerosing mucoepidermoid carcinoma with eosinophilia consistent with pathology from primary thyroid tumor. Histology shows (**a**) mucoid changes (hematoxylin and eosin (H&E) stain; original magnification, × 10), (**b**) epidermoid desmoplasia (hematoxylin and eosin (H&E) stain; original magnification, × 20), and (**c**) eosinophils present in inflamed stroma (hematoxylin and eosin (H&E) stain; original magnification, × 40)

One month after ^131^I ablation, the patient’s PET/CT scan revealed an interval development of a fluorodeoxyglucose avid 1.5-cm pulmonary nodule adjacent to left hilum within the left upper lobe and an 8 × 5-cm mass in the lower pole of the right kidney, which was biopsied (Fig. 1). The biopsy was morphologically consistent with metastatic SMECE (Fig. 2), and the tumor was also positive for *BRAF* V600E mutation. Two months after the ^131^I ablation, the patient received adjuvant external beam radiation. She received 54 Gy at 1.8 Gy per fraction to bilateral neck levels 2–6 along with superior mediastinal nodes. The thyroid bed, right neck levels 2–5, left neck levels 2–4, and peritracheal nodes went up to 60 Gy at 2 Gy per fraction. Repeat CT of the chest 1 year after initial presentation showed a new left suprahilar 3.2 cm × 2.3-cm mass with innumerable pulmonary nodules, increase in size of pleura-based density at the right lower lobe base of 3.8 × 1.1 cm, and left hilar lymphadenopathy. She presented several times for failure to thrive, which was thought secondary to the radical surgery. Her course was also complicated by acute renal failure and hematuria. Given rapid growth of metastatic lesions and declining functional status, she pursued hospice care and subsequently died within 1 year of diagnosis.

### Patient 2

A 73-year-old Caucasian woman with a history of PTC treated with total thyroidectomy at the age of 34 years presented to an outside institution with a recurrent right neck mass. She had not been routinely seen by any providers until this recurrence. She underwent right neck dissection, but the mass was found to be adherent to the carotid artery and esophagus, precluding complete resection. Pathology again revealed PTC. This was followed by treatment with 150 mCi of ^131^I 2 months postoperatively with subsequent whole-body scan uptake in the thyroid bed without evidence of distant metastasis. She was offered adjuvant external beam radiation to the neck but declined.

One year later, CT of the neck revealed a heterogeneously enhancing and partially necrotic mass within the right thyroidectomy bed extending posteriorly to the esophagus and involving the right recurrent laryngeal nerve. The mass measured 2.2 × 3.0 × 2.8 cm in its respective anterior-posterior, transverse, and craniocaudal dimensions. She was referred to our institution for surgical resection and underwent right radical neck dissection and wide local excision of the neck mass, though it was noted that residual tumor plaque on the carotid and trachea were unable to be fully resected.

**Fig. 3:**
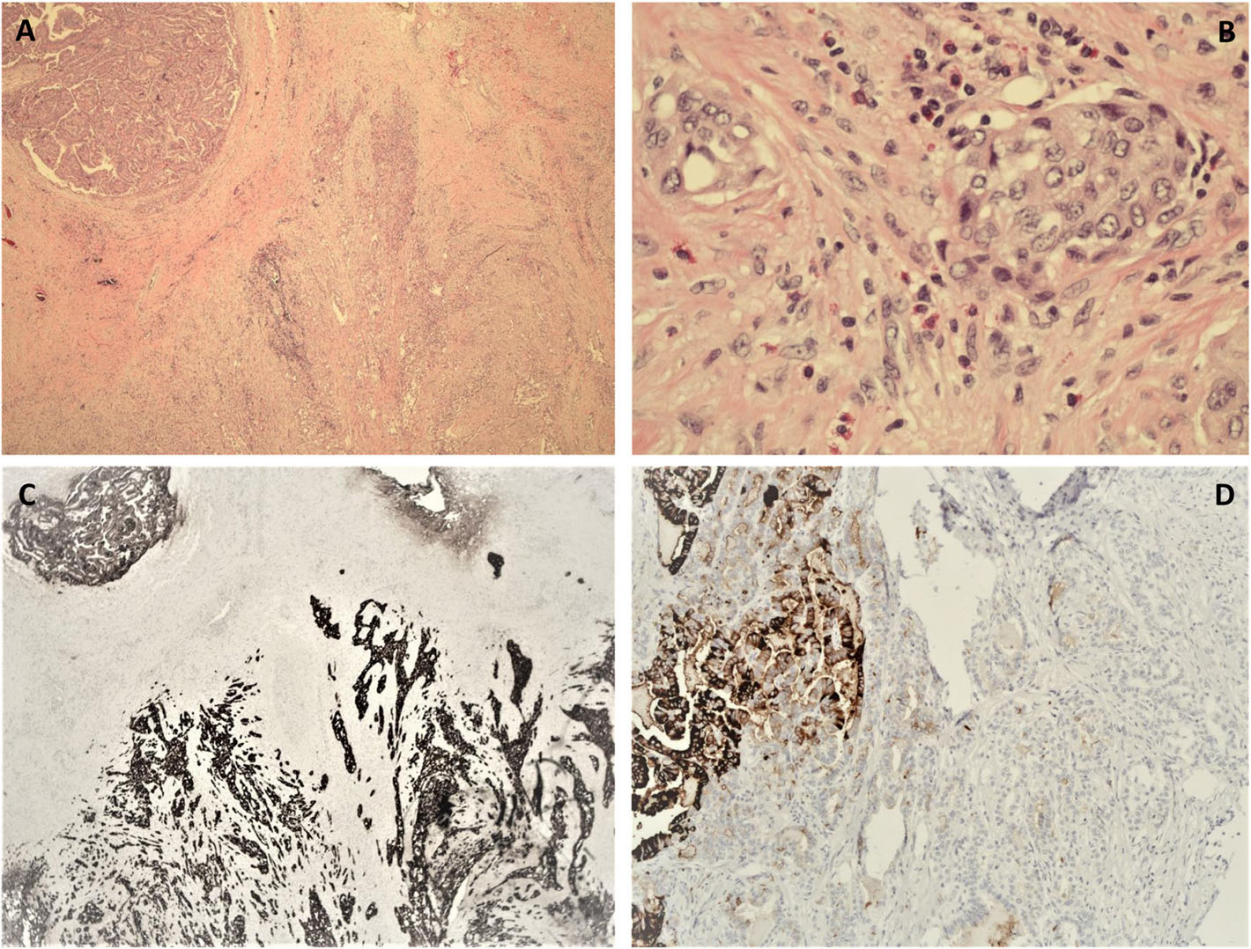
**a** Classic papillary thyroid carcinoma (PTC) on left upper corner and sclerosing mucoepidermoid carcinoma with eosinophilia (SMECE) on right lower corner (hematoxylin and eosin (H&E) stain; original magnification, × 2). **b** Nests of squamoid cells in a background of fibrous stroma and numerous eosinophils (hematoxylin and eosin (H&E) stain; original magnification, × 40). **c** Cytokeratin AE1/AE3 stain highlights classic PTC on left upper corner and infiltrating SMECE on right lower corner (IHC; original magnification, × 2). **d** Thyroglobulin stain is positive in classic PTC and negative in SMECE (IHC; original magnification, × 10)

Pathology revealed components of both classic PTC and SMECE. There was also a background of lymphocytic thyroiditis, and the tumor involved all margins, indicating that the tumor likely arose from a thyroid remnant. Upon immunohistochemistry, both PTC and SMECE stained positive for CK AE1/AE3 and negative for calcitonin. The PTC component stained positive for thyroglobulin, whereas SMECE was negative (Fig. 3). The SMECE-involved areas of the specimen were scattered to diffusely positive for CK5/6 and p63. *BRAF* V600E mutation was identified by PCR in both the PTC and SMECE tumors. The patient continued to follow up with her outside provider and had another treatment with ^131^I. Unfortunately, the dose of ^131^I administered and the post-therapy whole-body scan result were not available.

She did well until 11 months postoperatively, when she began to notice swallowing difficulty. Repeat CT of the neck revealed a mass in the region of the thyroid bed posterior to the trachea. These findings were confirmed on a PET scan. The patient underwent a right and left radical neck dissection with laryngectomy, though again the tumor was not able to be fully resected, because it was densely adherent to the carotid and innominate arteries. Surgical pathology showed anaplastic and poorly differentiated thyroid carcinoma, which again tested positive for *BRAF* V600E mutation. At the time of her last visit, the patient was being considered for radiation therapy and BRAF inhibitor treatment, but insurance did not cover the latter. She was subsequently lost to follow-up.

## Discussion and conclusions

To the best of our knowledge, these are the first published reports of SMECE associated with the activating mutation in the *BRAF* gene. *BRAF* V600E mutation is a novel independent molecular prognostic marker in the risk evaluation of thyroid cancer [8, 9]. It is associated with a poor clinical outcome with more aggressive, invasive tumors that are less ^131^I avid. This is consistent with the clinical presentation of both our patients. Patient 1 had highly aggressive metastatic disease and is the first reported case of SMECE with renal metastasis, to our knowledge. Patient 2 had locally invasive disease with multiple recurrences requiring repeated surgical interventions. Our findings are contrary to a recent paper that reported five patients with SMECE who did not have *BRAF* mutation by next-generation sequencing [2]. However, none of these cases had distant metastasis. Thus, although *BRAF* activating mutation may not be present in all SMECE thyroid cancers, it may be a marker for a subset of SMECE tumors that demonstrate more aggressive behavior, as seen in PTC.

Our literature review provides more insight into the characteristics of this rare thyroid cancer. We found 59 cases of SMECE reported in the literature, which are summarized in Tables 1 and 2 along with our 2 cases. Overall, there is a female predominance, with female-to-male ratio of 9:1. Patients ages ranged from 26 to 89 years with a median of 57 years. The mean tumor size, using the largest measured diameter reported, was 4.5 cm (range 0.5–13 cm). On initial presentation, the majority of tumors either occurred in the lateral lobes or diffusely involved the thyroid (98%), with fewer tumors occurring in the isthmus alone (2%). Almost all cases had a background of chronic lymphocytic thyroiditis (95%). We further observed that only seven cases (16%) had concurrent PTC, two of which were our cases. Thus, although coexisting SMECE and PTC is rare, it can occur. The majority of SMECE cases (95%) were negative for thyroglobulin, and all were positive for CK, p63, and mucin, whereas none stained for chromogranin or calcitonin. TTF-1 and CEA expression was more variable, with 47% and 75% of cases demonstrating expression, respectively.

**Table 1:** Literature review of sclerosing mucoepidermoid carcinoma with eosinophilia of the thyroid

Reference	Patient no.	Age (years)/sex	Location	Tumor size (cm)	Extrathyroid extension^a^	Lymph node metastasis^a^	Distant metastases^a^	Associated findings	Positive IHC^b^	Negative IHC^b^	Treatment	Additional treatment ^c^	Outcome
[1]	1	35/F	L	5.5	Present	None	None	LT	CEA, CK	Calcitonin, CG, TG	TT, RT	None	NED × 5.5 years
	2	64/F	L	3	None	None	None	LT	CEA, CK	Calcitonin, CG, TG	L lobectomy	None	NED × 1 year
	3	71/F	L	4.5	Present	None	None	LT	CEA, CK	Calcitonin, CG, TG	L lobectomy, isthmusectomy	TT, RT	NED × 3.5 years
	4	61/F	L	4	None	None	None	LT	CEA, CK	Calcitonin, CG, TG	L lobectomy	None	NED × 3 years
	5	43/F	Entire	NA	Present	None	None	LT	CEA, CK	Calcitonin, CG, TG	TT	RT	NED × 3 years
	6	46/F	NA	4	None	Present	None	LT, PTC	CEA, CK	Calcitonin, CG, TG	TT, LN diss	None	NA
	7	69/F	NA	7	Present	None	None	LT	CEA, CK	Calcitonin, CG, TG	TT	None	NA
	8	69/F	L	3	None	None	None	LT	CEA, CK	Calcitonin, CG, TG	TT	None	NA
[10]	9	57/F	Isthmus	1.2	None	None	None	LT	CK	Calcitonin, CG, TG	Isthmusectomy	None	NA
	10	46/F	Rt	2.3	None	None	None	LT	CEA, CK	Calcitonin, CG, TG	TT	None	NED × 8 years
	11	44/F	L	1.4	None	None	None	LT	CEA, CK	Calcitonin, CG, TG	L lobectomy	None	NED × 2 years
[11]	12	74/F	Rt	13	Present	Present	Bone, liver	LT	NA	Calcitonin, CG, TG	TT	None	Death × 2 weeks after TT
[7]	13	70/F	L	3	Present	Present	Bone, lung, subcutaneous tissue	LT	CEA, CK	Calcitonin, TG	TT, LN diss	CT, RT	AWD × 6 years
	14	69/F	Rt	2.5	Present	Present	None	LT, Rt lobectomy	CEA, CK	Calcitonin, TG	L lobectomy, neck diss	LN diss, RI, laryngopharyngectomy, esophagectomy, mediastinal diss, RT	NED × 12 years
[5]	15	39/F	Rt	NA	Present	Present	Lung	LT	Mucin	CEA, TG	TT, neck diss	CT	AWD × 4.5 years
	16	61/M	Rt	7.5	Present	Present	Bone, liver, peritoneum	None	Mucin	CEA, TG	TT, neck diss	RI, CT	AWD × 2 years
[12]	17	32/F	Rt	4	Present	NA	NA	LT	NA	NA	TT	None	NED × 14 months
[13]	18	57/F	Rt	5	Present	Present	None	LT	CEA, CK	Calcitonin, TG	TT	LN diss, neck diss, RT, laryngopharyngectomy	NED × 5 months
[3]	19	38/F	Rt	6	Present	Present	None	LT	CK	Calcitonin, TG	TT, neck diss	None	NED × 3 months
	20	47/F	Rt	5	None	None	None	LT	CK, mucin	Calcitonin, TG	Rt lobectomy	None	NED × 2 years
	21	73/F	Rt	3	None	None	None	LT	CK	Calcitonin, TG	Rt lobectomy	None	NA
	22	64/F	Rt	NA	None	None	None	LT	CK	Calcitonin, TG	Rt lobectomy	None	NA
[14]	23	39/F	Rt	6	Present	Present	None	LT, PTC	CK	Calcitonin, TG	TT, neck diss, LN diss, RT	None	NED × 5 years
[15]	24	38/F	Rt	4.8	Present	Present	None	LT	CK	Calcitonin, TG	TT, neck diss	None	NED × 3 years
	25	47/F	L	4.6	Present	Present	None	LT	CK	Calcitonin, TG	TT, neck diss	None	NED × 5 years
	26	52/F	L	2	None	None	None	NA	CK	Calcitonin, TG	TT	None	NED × 6 months
	27	45/F	L	3.5	Present	None	None	NA	CK	Calcitonin, TG	L lobectomy	Wide local excision	NED × 6 years
[16]	28	55/F	L	3.5	None	None	None	LT	NA	Calcitonin, TG	Subtotal thyroidectomy	None	NA
[17]	29	37/F	NA	NA	NA	NA	NA	NA	Mucin, p63	Calcitonin, TG, TTG	NA	None	NA
	30	57/F	NA	NA	NA	NA	NA	NA	Mucin, p63	Calcitonin, TG, TTG	NA	None	NA
	31	64/M	NA	NA	NA	NA	NA	NA	Mucin, p63	Calcitonin, TG, TTG	NA	None	NA
[18]	32	74/F	L	8	Present	None	None	LT	CK	Calcitonin, TG	RT	None	Death × 10 months
[6]	33	39/F	Rt	NA	Present	Present	Lung	LT	CK	TG, TTF, calcitonin	TT, neck diss	Neck diss, LN diss, RI, RT, CT	NA
[19]	34	65/F	Rt	4	None	Present	None	None	Mucin	Calcitonin, TG	Subtotal thyroidectomy	None	NA
[20]	35	59/F	Rt	4.5	None	None	None	LT	NA	NA	Rt lobectomy, neck diss	None	NA
[21]	36	55/F	Rt	9	Present	Present	None	LT, PTC	NA	NA	TT, neck diss	None	NA
[22]	37	45/M	Rt	1.5	None	None	None	LT	p63	CEA, calcitonin, TG, TTF	Rt lobectomy, isthmusectomy	None	NED × 6 years
[23]	38	52/F	L	4.6	None	None	None	LT	CK, p63, TTF	TG, calcitonin, CEA	TT	None	NED × 34 months
[4]	39	48/F	L	2.4	None	None	None	LT, PTC	p63	Calcitonin, TG, TTF	TT, RT	None	Alive
	40	45/F	Rt	NA	Present	Present	Lung	LT	p63, TTF	Calcitonin, TG	TT, neck diss, RT	Lung metastasectomy	Death × 3 years
	41	76/F	Rt	3.8	None	NA	NA	LT	p63, TTF	Calcitonin, TG	TT, RT	None	Death × 1.5 years
	42	89/F	Entire	NA	Present	Present	NA	NA	p63	Calcitonin, TG, TTF	TT, neck diss	None	Death × 8 years
	43	36/F	NA	NA	Present	Present	None	NA	P63, TG	Calcitonin, TTF	TT, neck diss, RT	None	Death
	44	71/F	Entire	10	Present	Present	Lung	NA	P63, TG	Calcitonin, TTF	TT, neck diss, RT	None	Death
[24]	45	26/F	NA	NA	NA		NA	NA	p63, TTF	NA	NA	None	NA
[25]	46	35/F	Rt	NA	None	Present	None	LT	CK, TTF	Calcitonin, TG	TT	None	NA
[2]	47	74/F	L	5	NA	NA	None	NA	p63	TG	Lt lobectomy	None	AWD × 4 years
	48	70/M	Rt	3	None	None	None	NA	p63	TG	TT	None	NA
	49	65/F	Rt	6	Present	Present	None	NA	p63	TG	Lobectomy, RT	None	Death × 1 year
	50	48/F	Rt	0.5	None	None	None	NA	p63	TG	Lobectomy	None	NED × 9 years
	51	30/M	Rt	0.5	None	NA	None	NA	p63	TG	TT	None	NA
	52	62/M	L	6	NA	NA	None	NA	p63	TG	TT	None	NA
	53	67/F	Rt	4	NA	None	None	NA	p63	TG	Rt lobectomy	None	NA
	54	77/F	Rt	6	NA	None	None	NA	p63	TG	TT	None	NED × 11 years
[26]	55	52/F	Rt	3.9	None	None	None	LT	NA	NA	Rt lobectomy	None	NED × 13 months
[27]	56	63/F	L	4.3	None	None	None	LT	CK, p63, TTF	TG, CEA	L lobectomy, LN diss, RT	None	NED × 20 years
	57	44/F	Rt	5.9	Present	None	None	LT, PTC	CEA, CK, p63, TTF	TG	TT, LN diss	None	NED × 3 years
	58	66/F	Rt	6.5	Present	None	None	LT	CEA, CK, p63, TTF	TG	TT, neck diss	None	NED × 18 months
[28]	59	58/F	L	5	Present	Present	None	None	TG, TTF	NA	TT, LN diss, RT	None	NA
Our patients	60	70/F	Rt	3	Present	Present	Lung, kidney	LT, PTC	CEA, CK	TG, TTF	TT, neck diss, laryngopharyngectomy, RI, RT	None	Death × 1 year
	61	74/F	Rt	3	Present	None	None	LT, PTC	CK, p63	Calcitonin, TG	Wide local excision, neck diss	RI, neck diss, laryngectomy	AWD × 3 years

*Abbreviations*: *F* female, *M* male, *LT* lymphocytic thyroiditis, *IHC* immunohistochemistry, *CK* cytokeratin, *TG* thyroglobulin, *CG* chromogranin, *CEA* carcinoembryonic antigen, *TTF* thyroid transcription factor-1, *NED* no evidence of disease, *AWD* alive with disease, *NA* not available, *RT* radiotherapy, *L* left, *Rt* right, *RI* radioiodine, *CT* chemotherapy, *TT* total thyroidectomy, *Diss* dissection, *PTC* papillary carcinoma of the thyroid, *LN* lymph node

^a^At time of presentation

^b^IHC evaluated for CK, CEA, TG, mucin, p63, TTF, CG, calcitonin

^c^Additional treatment refers to any subsequent therapy for local recurrence or metastatic disease

**Table 2:** Clinical and pathologic features of sclerosing mucoepidermoid carcinoma with eosinophilia of the thyroid

Feature	Data
Age	26–89 years (median 57)
Gender	55 F/6 M
Tumor size^a^	0.5–13 cm (4.5 cm); *n* = 49
Synchronous PTC	7/44 (16%)
Background of LT	42/44 (95%)
Extrathyroidal extension	28/52 (54%)
Lymph node metastases	20/50 (40%)
Distant metastases	8/54 (15%)
IHC:	
Cytokeratin	32/32 (100%)
Carcinoembryonic antigen	16/21 (76%)
Thyroglobulin	3/56 (5%)
Mucin	7/7 (100%)
p63	24/24 (100%)
Thyroid transcription factor-1	9/19 (47%)
Chromogranin	0/12 (0%)
Calcitonin	0/41 (0%)
Outcome data	
Alive without disease	25/40 (63%)
Alive with disease	6/40 (15%)
Deceased	9/40 (23%)

*Abbreviations*: *F* female, *M* male, *LT* lymphocytic thyroiditis, *IHC* immunohistochemistry, *PTC* papillary carcinoma of the thyroid

^a^Largest dimension of tumor used

Extrathyroidal extension and lymph node involvement of SMECE were present in 54% and 40%, respectively, at the time of presentation. Distant metastases were rare (15%), and sites included bone, liver, lung, peritoneum, and distant subcutaneous tissue, with the lung being most common. Patient 1 had renal metastasis showing SMECE pathology, which has never been reported. Aggregate outcome data of the case reports in our literature review revealed that 63% of patients were alive and free of disease, 15% of patients were alive with disease, and 23% of patients were deceased following initial diagnosis.

Both our patients had *BRAF* V600E mutation in the SMECE tumor tissue, suggesting involvement of the RAS-RAF-MEK-ERK signaling pathway in its pathogenesis. This observation opens potential treatment options for this poorly responsive thyroid cancer. We considered targeted therapy in the case of patient 1 but deferred it, given functional decline of the patient. In the case of patient 2, BRAF inhibitors were not covered by insurance. BRAF inhibitors such as vemurafenib and dabrafenib could be useful as targeted medical therapy in the treatment of SMECE. These medications have been approved by the U.S. Food and Drug Administration for the treatment of metastatic melanoma [29, 30]. They have also shown antitumor efficacy in progressive, *BRAF* V600E mutant papillary, and anaplastic thyroid cancer when combined with a MEK inhibitor [31, 32]. One limitation of our analysis is the mechanism by which *BRAF* V600E mutation was detected. PCR was used because newer techniques of molecular sequencing, such as next-generation sequencing, were not widely available at the time of these patients’ presentations.

In conclusion, we report the first two cases of SMECE associated with activating *BRAF* mutation. These findings demonstrate that these tumors should be tested early for *BRAF* mutation and provide insight into potential mechanisms of the pathogenesis of aggressive subtypes of SMECE. BRAF inhibitors are currently being investigated for use in thyroid cancers as targeted pharmacotherapy and may also prove to be useful in the treatment of a subset of SMECE thyroid cancer.

## Data Availability

Not applicable.
